# New-Onset Headache and Abnormal Eye Movements in a Four-Year-Old Child: Indicators of Increased Intracranial Pressure

**DOI:** 10.7759/cureus.26850

**Published:** 2022-07-14

**Authors:** Sarah C Miller, Carl E Stafstrom

**Affiliations:** 1 Neurology, Johns Hopkins University School of Medicine, Baltimore, USA

**Keywords:** papilledema, headache in children, 6th nerve palsy, pseudotumor cerebri, idiopathic intracranial hypertension

## Abstract

A four-year-old previously healthy child presented with new-onset, diffuse, severe headache, and left sixth nerve palsy. The child was evaluated at several acute care facilities, at which the symptom of "crossing eyes" was not addressed specifically. At our emergency department, on day 6 of symptoms, a left cranial nerve 6 palsy was diagnosed; on brain MRI scan, there was evidence of increased intracranial pressure (distended optic nerve sheaths, flattened posterior sclerae), which was confirmed by lumbar puncture, which showed an opening pressure of >36 cm H_2_O. Idiopathic intracranial hypertension (IIH) was diagnosed, and all symptoms abated with two months of treatment with acetazolamide. IIH should be considered in a child with headache and abnormal eye movements.

## Introduction

Headache is a common symptom in children presenting for emergency care [1-2]. However, in children, headaches are often challenging to categorize due to the difficulty of young patients to effectively communicate their symptoms as well as the numerous etiologies that can lead to similar presentations. These challenges are compounded when they occur in the context of other signs of acute illness. Visual symptoms, including abnormal eye movements, are an important indicator of a potentially serious neurological disease that presents with a headache in children. To diagnose such conditions accurately, a complete description of visual symptoms is critical to assess for possible intracranial pathology. The child discussed here was diagnosed correctly only after presenting to other acute care facilities several times. We emphasize the critical role of characterizing visual symptoms to guide appropriate investigations and treatment.

## Case presentation

A four-year-old, non-obese child with normal development and no significant past medical history presented to the primary care provider for a severe headache. The patient’s mother reported that symptoms had started three days prior to presentation when the child first experienced nausea, vomiting, abdominal pain, and chills in the context of a severe, left temporal, non-positional headache. A rapid streptococcal pharyngitis test was equivocal but the child was prescribed amoxicillin. Later the same day, the patient presented to an emergency department with persistent symptoms, received an antiemetic, and was sent home. Earlier that day, the mother had noted changes in the child’s eye movements, which she described as the eyes “crossing.” She was unable to provide further details but reported that her child’s “eyes seem to be looking toward the nose.” On day 6 of illness, the patient presented to a second emergency department for the chief complaint of persistent headache and eye movement changes; the child was prescribed acetaminophen and was again discharged without further workup. Later that night, the patient presented to the Johns Hopkins (JH) pediatric emergency department.

The child had no history of headache, trauma, or relevant familial disease. There was no recent fever, rash, diarrhea, or constipation but poor food and drink intake was endorsed since symptom onset. The child had not experienced any recent gait or coordination changes or focal neurologic deficits other than abnormal eye movements. The child had been receiving acetaminophen twice daily for the headache, without improvement.

In the JH emergency department, the child denied a pressure feeling or ringing in the ears and denied double vision but was observed to cover the left eye while watching television. A head CT without contrast was read as normal, ruling out a mass or hydrocephalus. Examination by a consultant ophthalmologist revealed normal tonometry, 20/20 visual acuity in both eyes, equal, round, reactive pupils, slightly decreased abduction of the left eye (left cranial nerve 6 palsy), esotropia with intermittent in-turning of the left eye (exacerbated when looking near), normal color vision, and normal funduscopic examination with no evidence of papilledema. In the emergency department, the child was noted to be bradycardic (54-68 beats per minute) and intermittently hypertensive (115-120/63-73).

**Figure 1:**
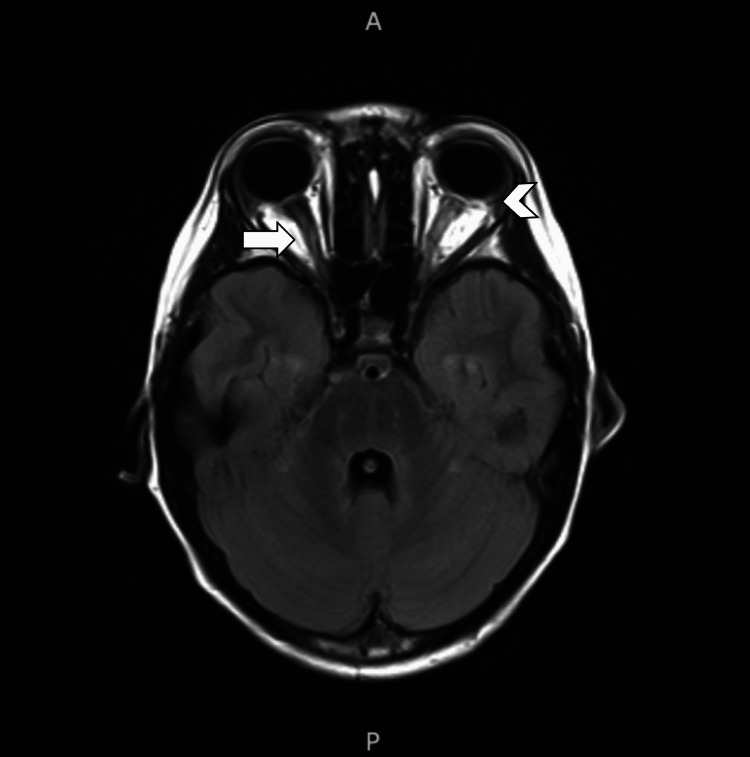
Brain MRI scan Demonstration of increased intracranial pressure on T1-weighted brain MRI scan, showing optic nerve sheath distension (arrow) and flattening of the posterior globes (arrowhead)

Out of concern for acute neurologic change, as indicated by the left cranial nerve 6 palsy, the patient was admitted to the hospital for further work-up. Complete blood count and comprehensive metabolic panel were normal. A brain MRI scan ruled out intracranial hemorrhage and mass, and the cisternal segments of the cranial nerves were normal. However, there was distention of both optic nerve sheaths and flattening of the posterior globes (Figure 1), suggestive of increased intracranial pressure (ICP) despite the lack of papilledema on funduscopic examination. The child underwent a lumbar puncture, showing a markedly elevated opening pressure of >36 cm H_2_O. Approximately 35 mL of cerebrospinal fluid (CSF) were removed, with a closing pressure of 16 cm H_2_O. The CSF had slightly low protein but was otherwise normal, including gram stain, bacterial and viral (herpes simplex virus (HSV), Epstein-Barr virus (EBV), varicella-zoster virus (VZV)) cultures, and Lyme antibody. Based on the elevated opening pressure without an identifiable structural or infectious etiology, the patient was diagnosed with idiopathic intracranial hypertension (IIH, also known as pseudotumor cerebri). The patient was discharged on acetazolamide 75 mg twice daily with the plan of close follow-up by pediatric neurology and ophthalmology.

When the patient was seen four weeks after discharge, there was a complete resolution of headaches and visual symptoms, with normal eye movements, no cranial nerve 6 palsy, and no evidence of papilledema. Blood pressure (102/56) and heart rate (90 beats per minute) had returned to normal. Two months after discharge, acetazolamide was tapered, and the child remains headache-free without visual symptoms eight months after the initial presentation.

## Discussion

Headaches are a common cause of children’s presentation to emergency departments. The majority of headaches in children have benign etiologies such as primary headaches (migraines or tension-type headaches) [1]. These primary headaches are usually episodic and have a subacute to gradual onset [2]. Migraines may be associated with phonophobia, photophobia, nausea, and vomiting [2]. The International Classification of Headache Disorders provides criteria for differentiating primary headaches from secondary headaches [3]. However, some presenting symptoms imply potentially serious etiologies, especially if there are concurrent neurological changes and acute onset [2]. Certain headache features, such as positionality, progressive nature, unilaterality, or occipital localization, are more concerning for an underlying cause [2].

IIH consists of increased ICP without an identifiable etiology, such as hydrocephalus or mass, in the context of normal CSF characteristics; neurological imaging can be normal or show signs of increased intracranial pressure such as those seen in our patient - flattened posterior globes or distended optic nerve sheaths [4-5]. IIH has an incidence of 0.9 per 100,000 adults and 0.63 per 100,000 children [4-6]. Symptoms are most commonly secondary to elevated ICP and consist of headache, nausea, vomiting, and occasionally Cushing’s triad of bradycardia, bradypnea, and hypertension [7]. Decreased lateral eye movements as a manifestation of cranial nerve 6 palsy from increased intracranial pressure is a characteristic finding in IIH. Cranial nerve 6, which mediates lateral eye movements, is readily compressed over its long intracranial course from the caudal pons to the lateral recti muscles, causing inwardly turning eyes and limitation of abduction. Unilateral sixth nerve palsies are common in this context but children do not always complain of subjective diplopia. Indeed, pediatric patients, especially those under seven years of age, often do not even complain of headaches, at least initially, in IIH [8]. In this young population, it is important to examine vision and eye movements carefully to rule out IIH.

For children presenting with headaches in general, a wide differential diagnosis must be considered, and a full headache history must be obtained. It may be difficult for young children to characterize their headache symptoms in words, due to incomplete understanding and insufficient vocabulary. For example, children may not be able to characterize their headache as focal or diffuse or to recognize and explain associated signs and symptoms such as nausea, diplopia, visual blurriness, or ophthalmoplegia [2]. It is therefore advisable that providers probe with questions related to headache character as well as carefully observe the child’s behavior (e.g., preferring a dark room or covering their head with a blanket infers photophobia, or in our case, covering one eye to see more clearly, ostensibly by reducing diplopia).

In addition, the physical examination may not fully capture neurologic findings in young patients with headaches [2,9]. This may explain, at least partially, how pediatric patients with IIH have been more likely to be described as “asymptomatic” at diagnosis than adults, as visual changes are often seen secondary to IIH and may be underdiagnosed in this population [4,10]. For this reason, for young patients who demonstrate any warning signs, such as acute onset, physicians may employ age-appropriate techniques to ensure that any visual changes are documented. One useful technique is to ask the child to draw a picture of their headache - where it is located, how it makes them feel, and whether there are associated visual changes or other symptoms. We have shown that children’s headache drawings that depict evidence of diplopia (such as seeing/drawing double or depicting overlapping images) correlates highly with IIH and not with migraine [9]. Identification of visual changes is critical because routine eye exams are often the first line of detection for patients with IIH through the visualization of papilledema or optic disc swelling [5,10]. However, up to 48% of pediatric IIH patients do not have papilledema visible by funduscopic exam such as our patient [11]. By comparison, 95% of adults with IIH develop visibly identifiable papilledema by the time of diagnosis [11]. It must be acknowledged that vision and eye movement abnormalities can be subtle and take some time to develop in IIH [2,4].

For these reasons, physical examination findings and vital signs should be monitored carefully in the evaluation of a child with headaches and vomiting. Further, neurological changes in the context of this presentation should raise alarm for an intracranial etiology. Despite our patient having a relatively characteristic presentation of IIH, including heralding symptoms, such as headache and vomiting, signs of increased ICP with hypertension and bradycardia, and eventually, acute eye movement limitations indicative of cranial nerve 6 involvement, our patient presented four times to healthcare facilities before receiving a neurological work-up and the correct diagnosis.

The acute resolution of our patient’s symptoms was likely related to the ameliorative effects of the lumbar puncture, with a marked reduction of ICP after removal of CSF. For chronic treatment, acetazolamide, a carbonic anhydrase inhibitor, reduces CSF production by the choroid plexus, lessening the chance of recurrence of elevated ICP in patients with IIH [4]. Topiramate, another carbonic anhydrase inhibitor, is gaining popularity as an IIH therapy, but there are no randomized clinical trials and long-term cognitive effects can be problematic [12]. In obese patients, weight loss has been shown to be beneficial. The goal of any IIH treatment is to reduce acute symptoms (headache, diplopia) and avert vision loss [4,12].

## Conclusions

IIH is a rare but critical diagnosis to consider when a child presents with headaches and focal neurological signs such as eye movement abnormalities. While it is important to consider infectious causes of headache and vomiting, as they are extremely common in children, providers must keep differential diagnoses broad before excluding “can’t miss” diagnoses such as IIH.
